# Supplementary material for: Granulins Regulate Aging Kinetics in the Adult Zebrafish Telencephalon
Source: Cells. 2020 Feb 3;9(2):350. doi: 10.3390/cells9020350 (PMC7072227; doi:10.3390/cells9020350)
Supplement: Supplementary file 1 [file cells-09-00350-s001.zip › Figure S6_Heat maps of genes associated with the main GO terms described in Figure 8.pdf]

Notch signalling pathway

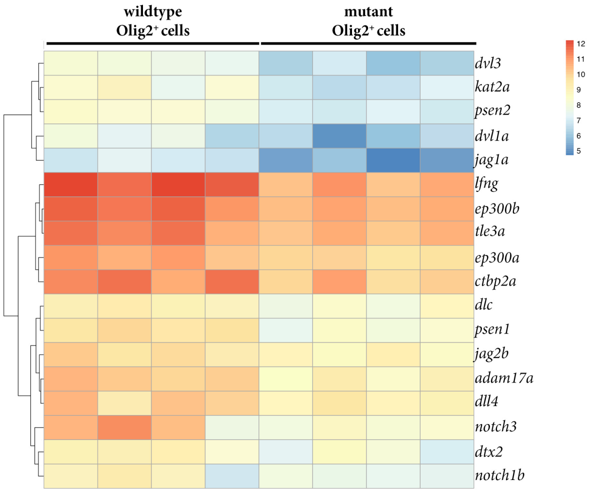

Hedgehog signalling pathway

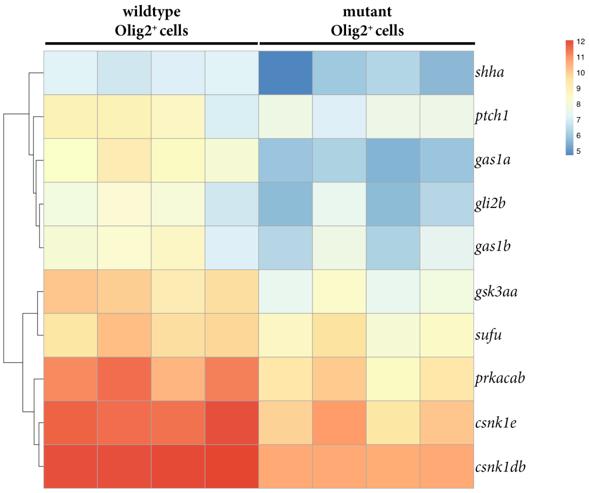

ErbB signalling pathway

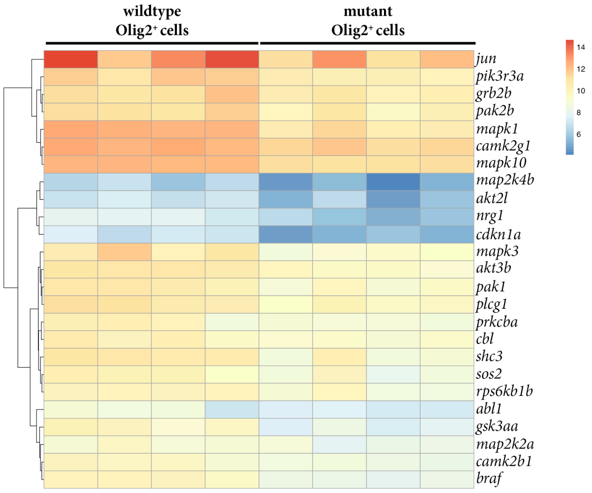

Negative regulation of BMP signalling pathway

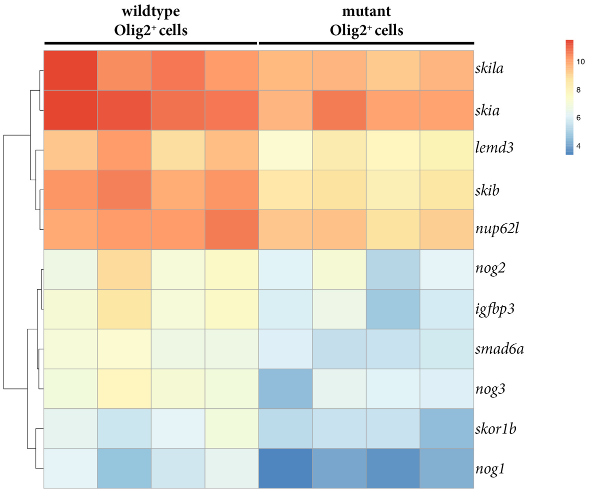

TGF-beta signalling pathway

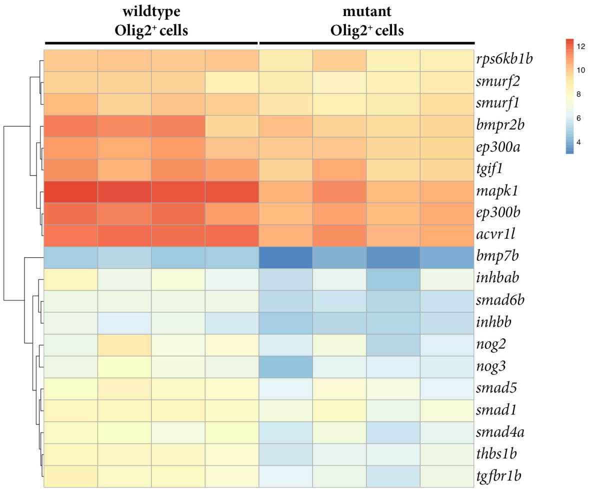

Zambusi et al, 2019; Figure Suppl. 6
