# Supplementary figures and images for: Granulins Regulate Aging Kinetics in the Adult Zebrafish Telencephalon
Source: Cells. 2020 Feb 3;9(2):350. doi: 10.3390/cells9020350 (PMC7072227; doi:10.3390/cells9020350)

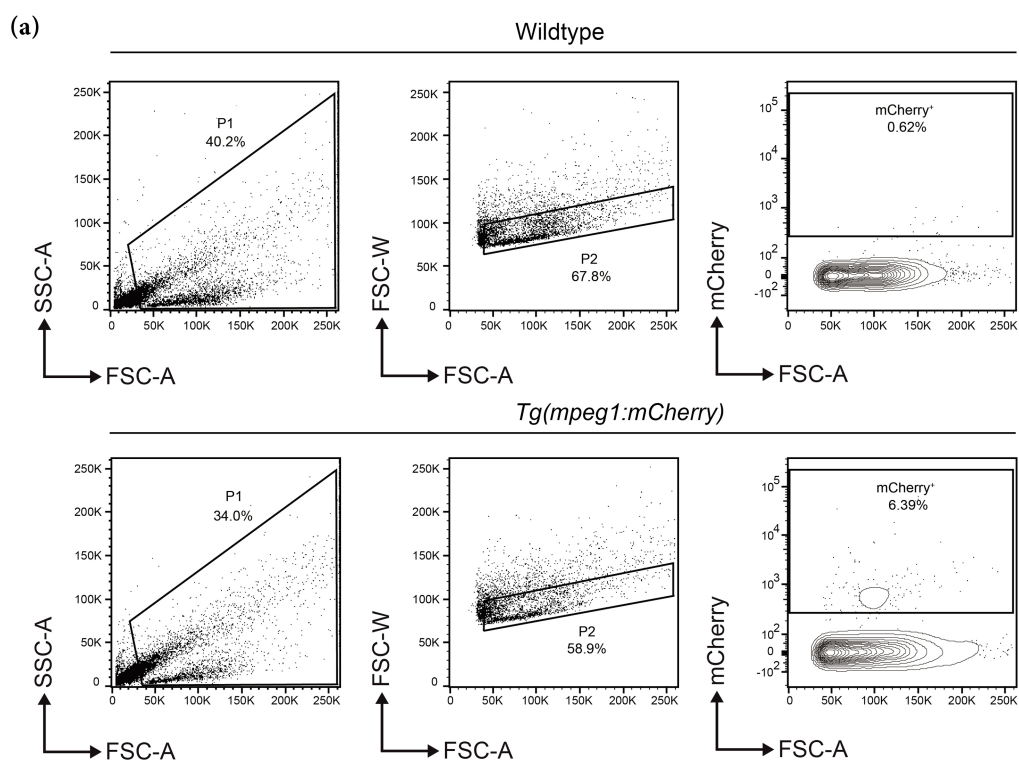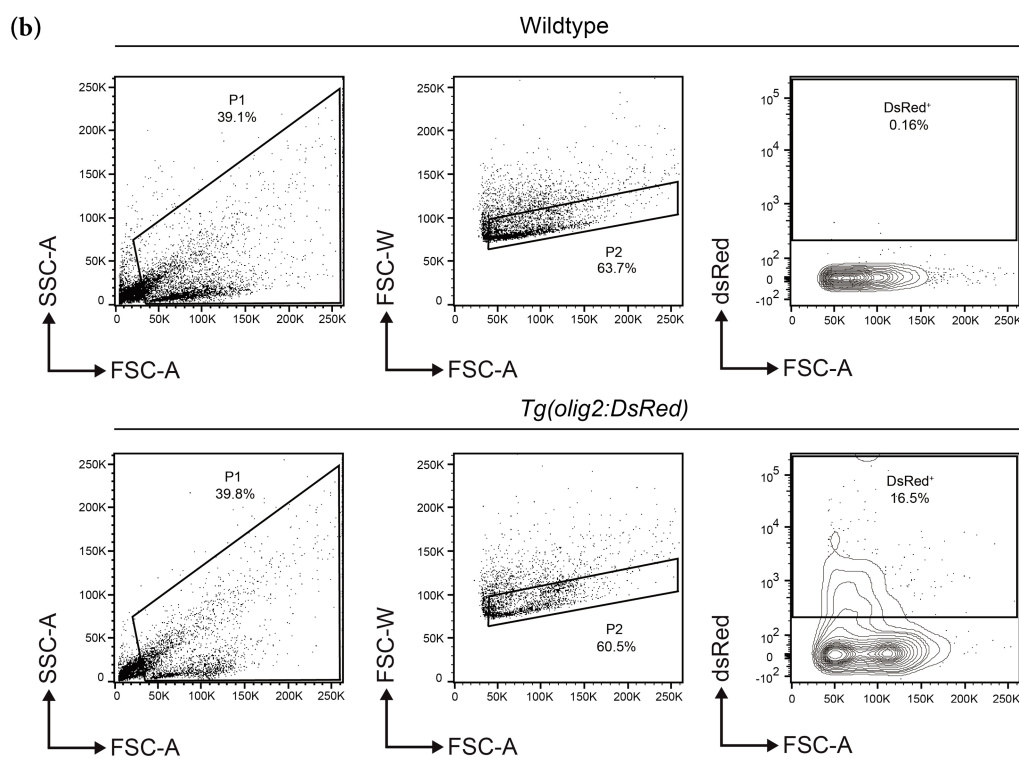

Supplement: Supplementary file 1 [file cells-09-00350-s001.zip › Figure S1_FACS plots illustrating sorting gates.pdf]

(a)

FACS isolated Mpeg1<sup>+</sup> cells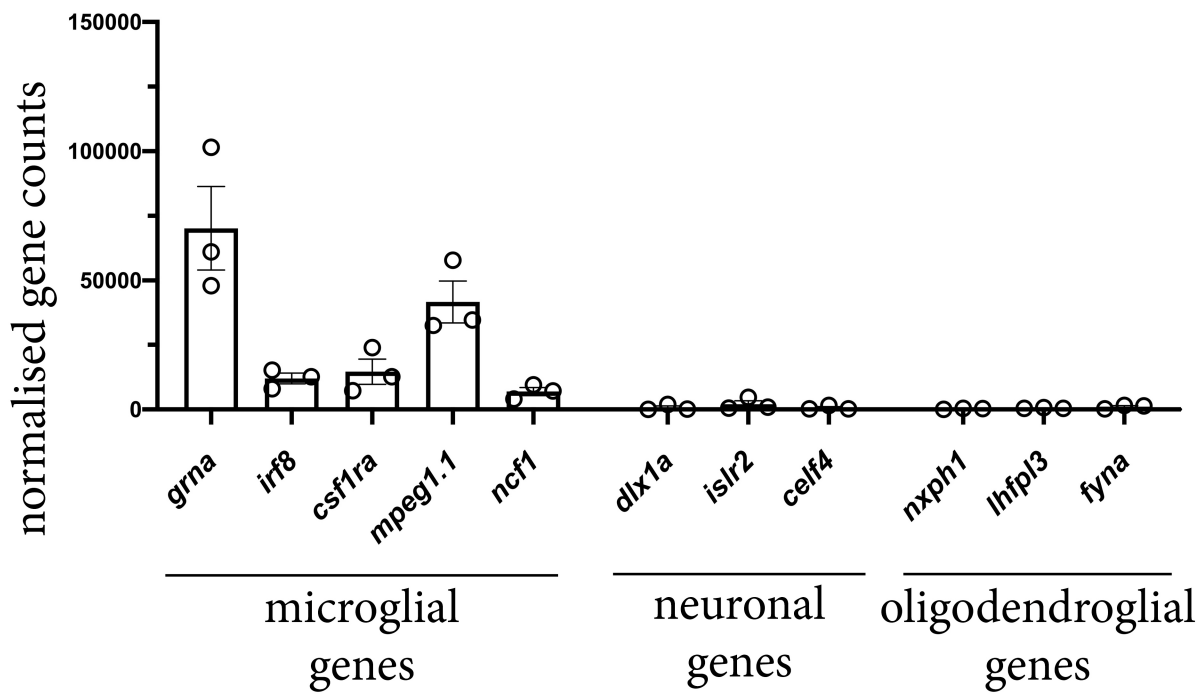

(b)

FACS isolated Olig2<sup>+</sup> cells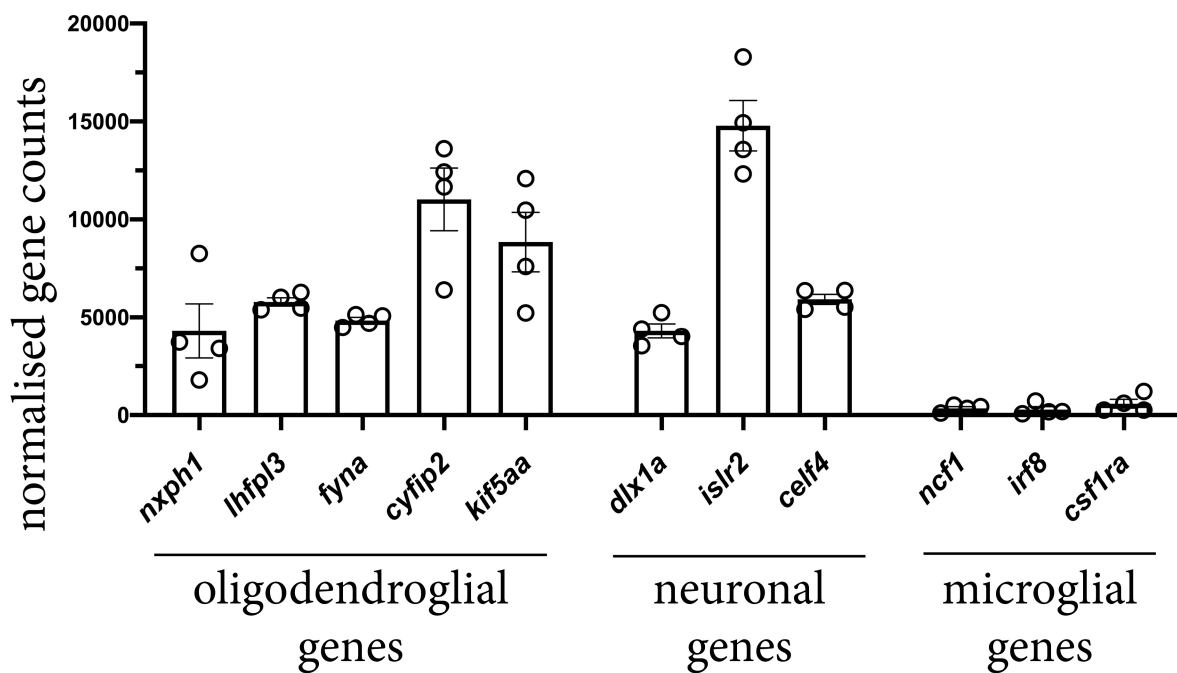

(c)

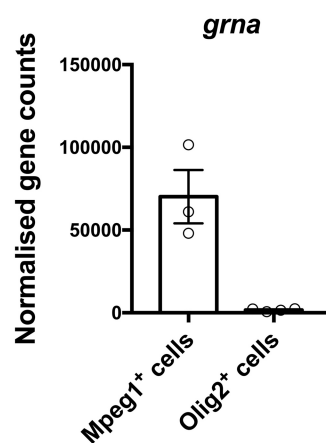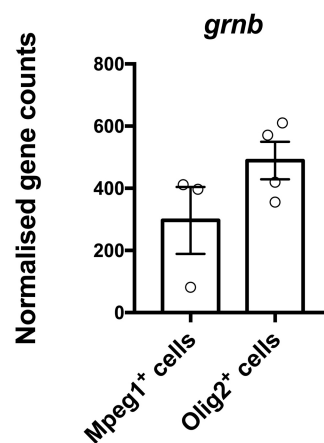

Supplement: Supplementary file 1 [file cells-09-00350-s001.zip › Figure S2_Enriched genes in FACS sorted populations.pdf]

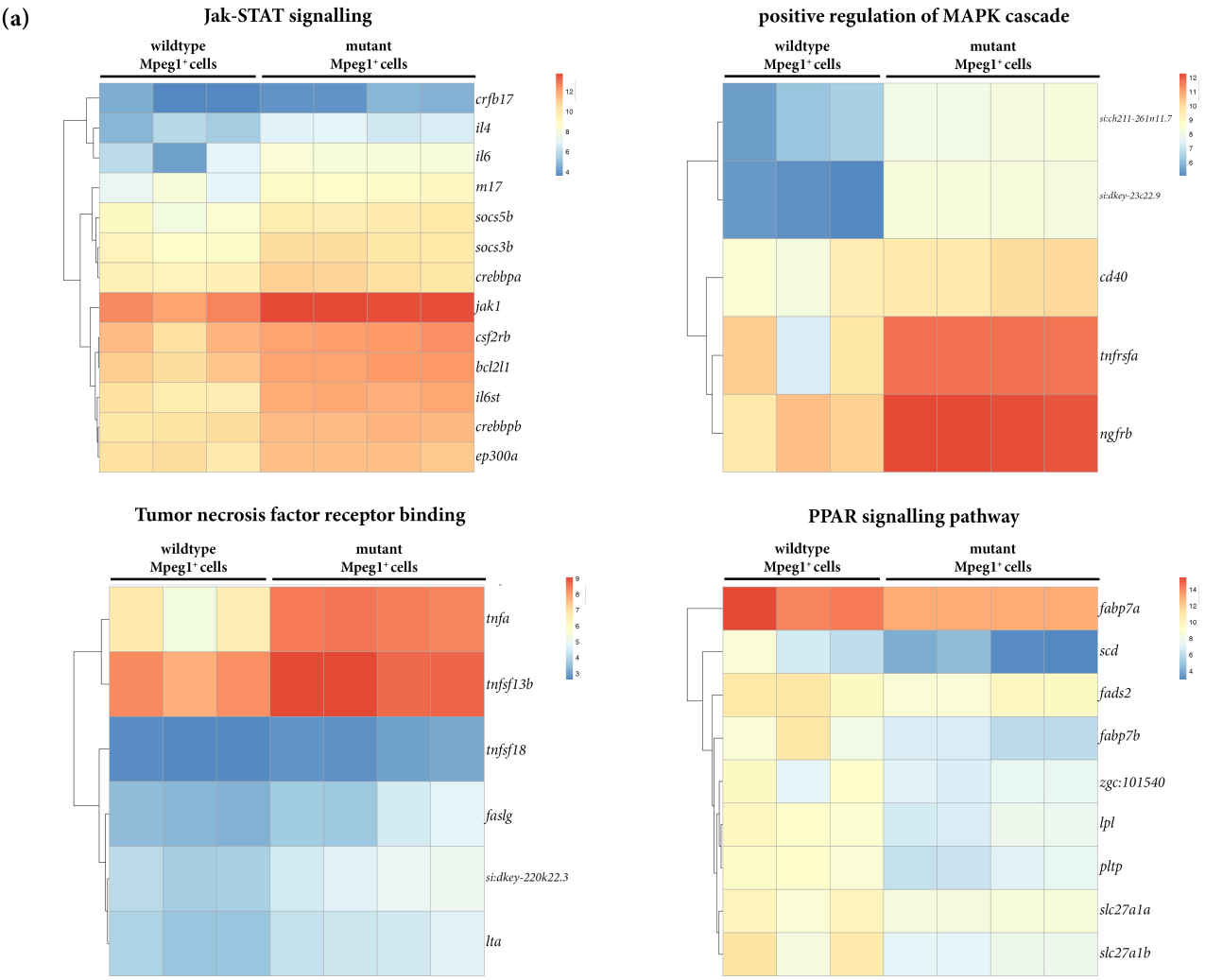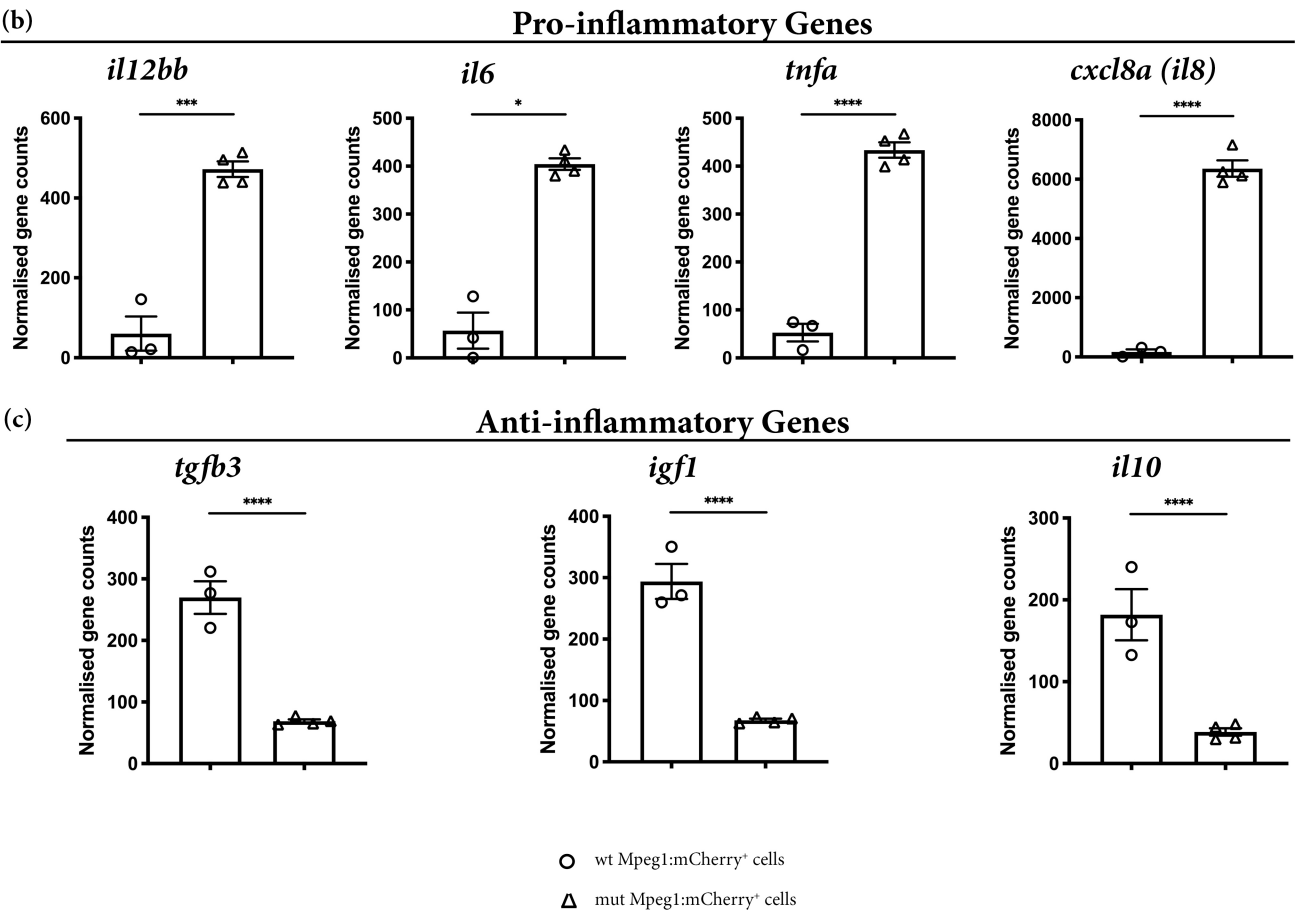

Supplement: Supplementary file 1 [file cells-09-00350-s001.zip › Figure S3_Heat maps of genes associated with the main GO terms described in Figure 1 and mRNA levels of pro-inflammatory and anti-inflammatory cytokines in wildtype and mutant microglial cells.pdf]

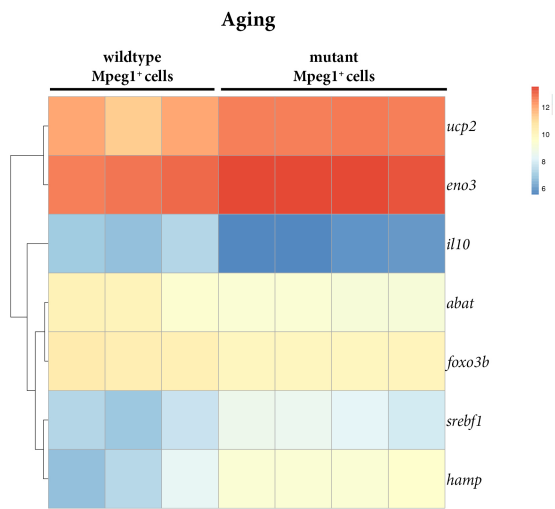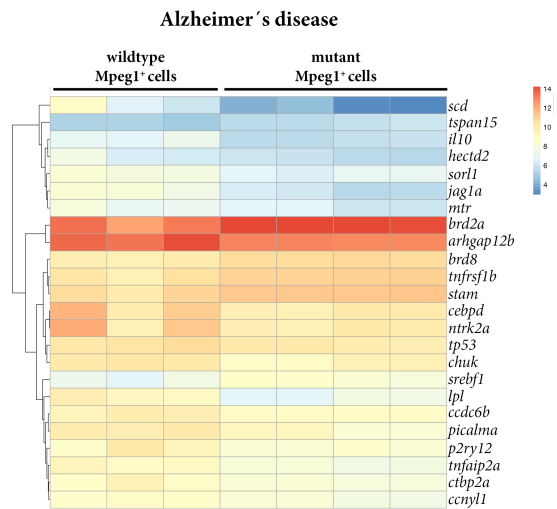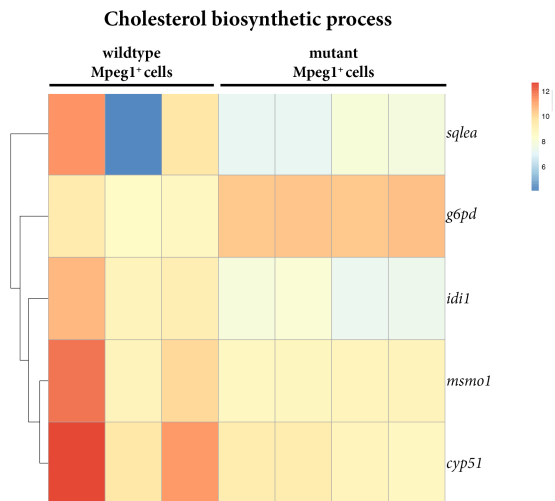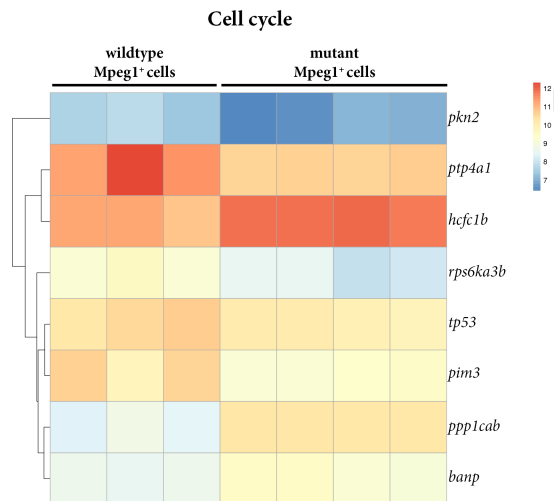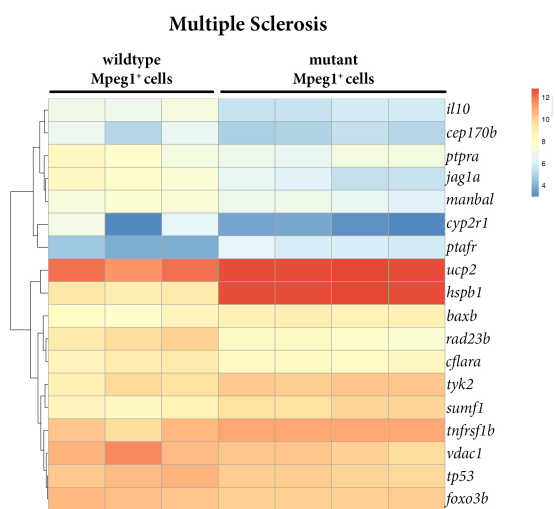

Supplement: Supplementary file 1 [file cells-09-00350-s001.zip › Figure S4_Heat maps of genes associated with the main GO terms described in Figure 3.pdf]

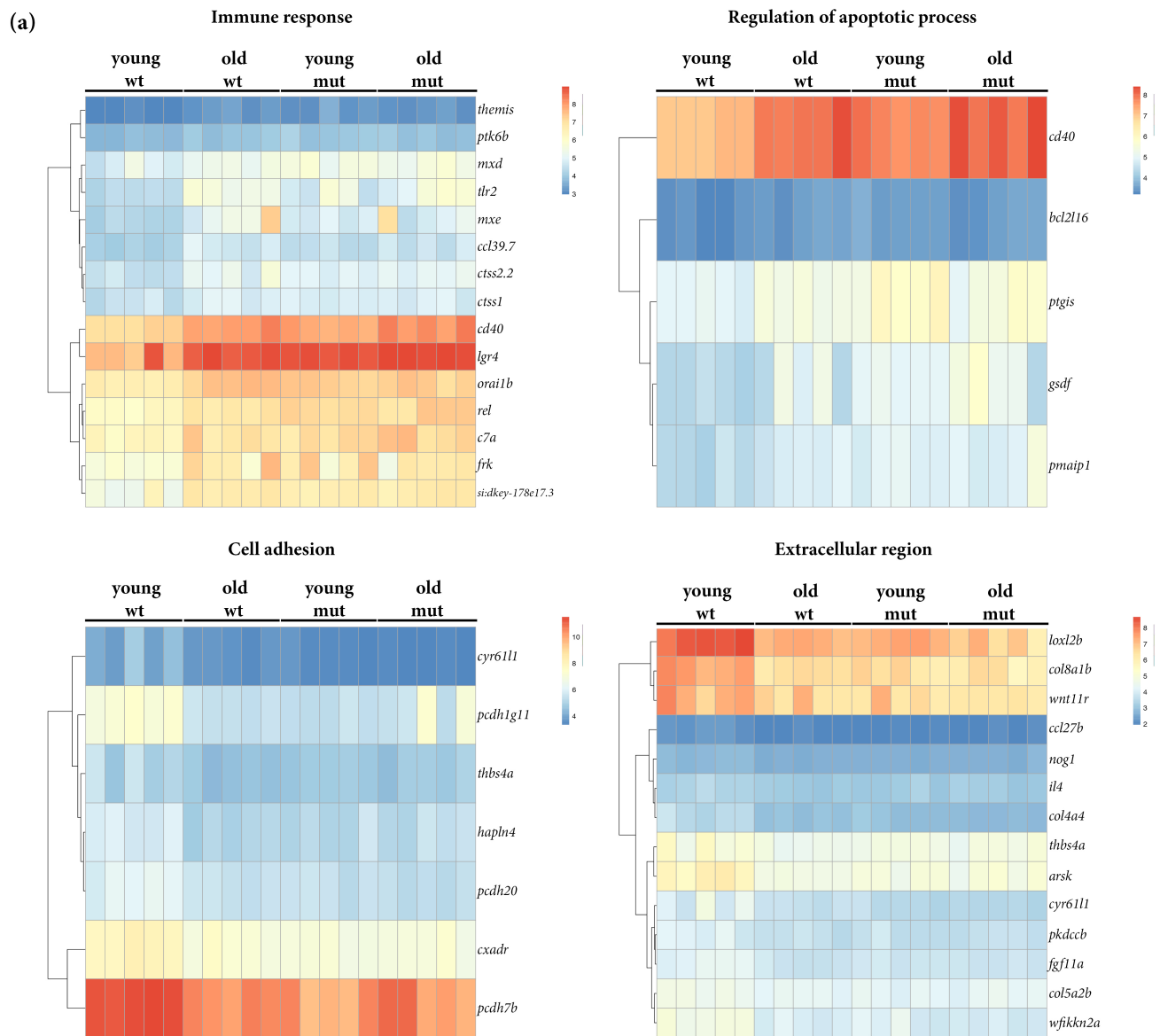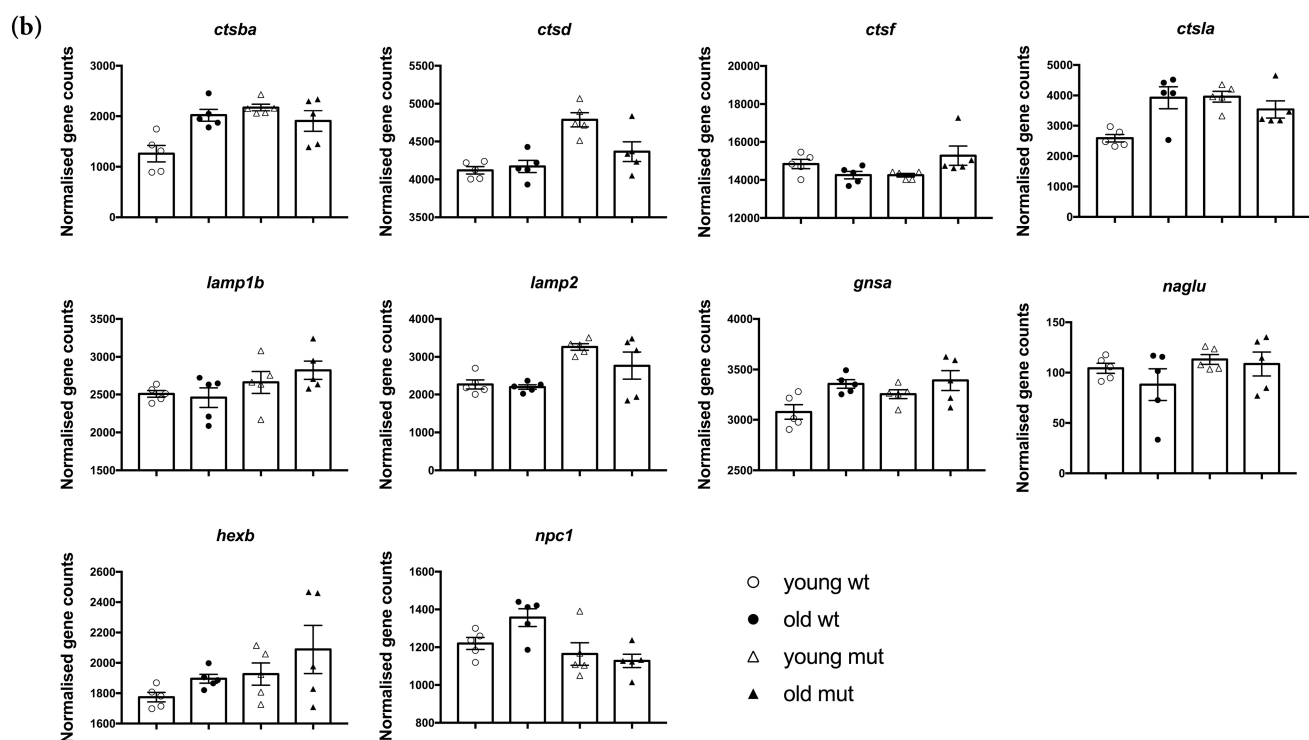

Supplement: Supplementary file 1 [file cells-09-00350-s001.zip › Figure S5_Heat maps of genes associated with the main GO terms described in Figure 5 and mRNA levels of lysosomal genes in the whole telencephalon.pdf]
